# Genomic Characterization of Extended-Spectrum *β*-Lactamase (ESBL) Producing *E. coli* Harboring *bla*_OXA−1_-*catB3-arr-3* Genes Isolated From Dairy Farm Environment in China

**DOI:** 10.1155/2024/3526395

**Published:** 2024-10-11

**Authors:** Muhammad Shoaib, Minjia Tang, Furqan Awan, Amjad Islam Aqib, Ruochen Hao, Saad Ahmad, Shengyi Wang, Ruofeng Shang, Wanxia Pu

**Affiliations:** ^1^Key Laboratory of New Animal Drug Project, Gansu Province/Key Laboratory of Veterinary Pharmaceutical Development, Ministry of Agriculture and Rural Affairs/Lanzhou Institute of Husbandry and Pharmaceutical Sciences of Chinese Academy of Agricultural Sciences, Lanzhou 730050, China; ^2^Department of Epidemiology and Public Health, University of Veterinary and Animal Sciences, Lahore 54000, Pakistan; ^3^Department of Medicine, Cholistan University of Veterinary and Animal Sciences, Bahawalpur 63100, Pakistan

**Keywords:** bioinformatics analysis, *bla*_OXA−1_-*catB3*-*arr-3* gene array, dairy farm environment, *Escherichia coli*, whole genome sequencing

## Abstract

Anthropogenic activities in the environment affect the ecosystem and can play an important role in selecting and spreading antibiotic-resistant bacteria (ARB) and genes (ARGs). The dairy farm environment may serve as a hotspot and reservoir for exchanging and spreading ARGs, but studies are scarce. Here, we investigated and characterized the extended-spectrum *β*-lactamase producing *Escherichia coli* strains recovered from the dairy farm environment co-harboring *bla*_OXA−1_, *catB3*, and *arr-3* genes. The isolates were identified and characterized by PCR, antimicrobial susceptibility testing, conjugation assay, whole genome sequencing (WGS), and multiple bioinformatics tools. Seven *E. coli* strains co-harboring *bla*_OXA−1_, *catB3*, and *arr-3* genes were identified which belonged to distinct sequence types (STs) and carried diverse plasmid replicon types. The conjugation assay revealed a successful transfer of *bla*_OXA−1_, *catB3*, and *arr-3* genes into the recipient *E. coli* J53 with a co-conjugation frequency ranging from (2.25 ± 0.3) × 10^−4^ to (3.85 ± 0.3) × 10^−3^. Bioinformatics analysis of WGS revealed the diversity of acquired ARGs, conferring resistance to aminoglycosides, beta-lactams, quinolones, tetracyclines, macrolides, trimethoprim–sulfamethoxazole, phosphonic, phenicol, and rifamycin. The genetic environment analysis showed that *aac*(*6′*)-*Ib-cr-bla*_OXA−1_-*catB3-arr-3-qacE1-sul1* was the common genetic backbone among the seven *E. coli* strains. Among the mobile genetic elements, insertion sequences were the predominant elements as compared to transposons. The phylogenetic analysis demonstrated a close relationship between the *E. coli* of this study and other strains of human–animal-environment origin retrieved from the NCBI database. This study presented the whole genome-based characterization of *E. coli* strains carrying the *bla*_OXA−1_-*catB3-arr-3* genes. It provided evidence that the dairy environment may harbor a variety of ARGs and act as a potential reservoir for their spread in the ecosystem. The results recommend the routine surveillance of ARGs carrying bacteria in dairy environments and the need for additional studies to understand the dissemination mechanism within One Health perspective to prevent their further spread.

## 1. Introduction

According to the World Health Organization (WHO), antimicrobial resistance (AMR) is known among the top 10 threats to global public health [1]. AMR not only contributes to increased death rates but also affects the economics of any country due to increased therapeutic costs in response to resistant bacterial infection. It is documented that only in 2019, the deaths of people reached 1.2 million globally because of antibiotic-resistant bacteria (ARB) [2]. Nowadays, the increase in the development of AMR and its environmental expansion is a chief concern for public health. Consequently, to direct research, exploration, and production of new drugs, WHO released a worldwide priority list of significant ARB [3]. The extended-spectrum *β*-lactamase (ESBL)-producing *Enterobacteriaceae*, such as *Escherichia coli*, fall under first-priority pathogens of critical importance [4]. In recent years, it has become clear that the spread of ESBL-producing *E. coli* is not only limited to the clinical and healthcare sectors but also extends to environments with anthropogenic or agricultural impact [5].

Livestock species and the surrounding environment are also the carriers of ESBL-producing *E. coli* as it is a commensal bacterium colonizing the gut of animals and a potential reservoir of carrying multiple antibiotic resistance genes (ARGs) because of the extensive use of antibiotics in agriculture and animal farming [6]. Most AMR studies are limited to humans and animals, but the occurrence and spread of AMR determinants need a fully integrated approach at the animal, human, and environmental interface [7]. The role of the environment in the selection and spread of ARB and ARGs, known as biotic effluence, is well recognized, particularly in anthropogenically affected ecosystems, and is considered an essential ecological issue [8, 9]. Previously, researchers have emphasized the role of the natural environment and how freshwater bodies are particularly susceptible to antibiotic contamination from various sources such as sewage, hospital, domestic, and municipal wastes [5, 10–12]. The discharge of animal waste into the environment also aggravates the transmission of ARGs harbored by ARB through horizontal gene transfer (HGT) and selective pressure among the strains of the same or different species [13–15]. Thus, the presence of antibiotics, nutrients, and bacterial populations in the environment provides an ideal condition for the reproduction and development of unique antibiotic resistance patterns and, concurrently, the emergence of MDR bacteria, which pose a significant threat to public health [12, 16, 17].

Multiple previous studies highlighted the role of animal-associated *E. coli* in transmitting ARGs from animals to humans through different routes [18–20]. Here, we reported the co-emergence of *bla*_OXA−1_, *catB3*, and *arr-3* genes conferring resistance to beta-lactam, chloramphenicol, and rifampicin (RIF), respectively, because it has been least or not reported previously in *E. coli* strains isolated from a dairy farm environment. RIF is an important first-line drug used in human medicine as an antituberculosis with isoniazid [21]. RIF is also commonly used to treat prosthetic joint infections caused by multiple pathogens because of its excellent penetration and biofilm-degrading ability [22]. Combined with meropenem (MEM), RIF also effectively treats MDR *Acinetobacter baumannii* infections [23]. Chloramphenicol was an extensively used drug in China before 1999. However, chloramphenicol resistance genes (*cat*) remain in the environment. This may be due to the HGT of genes within and between the bacterial species [24]. Later, chloramphenicol was replaced with another derivative, i.e., florfenicol, which was found quite effective in the control of bovine respiratory disease, foot rot, and intracellular pathogens because of its excellent penetration across the blood–brain barrier [25, 26]. Similarly, beta-lactams are also extensively used drugs in human medicine worldwide [27] as well as in livestock [28] and account for 60% of all antibiotics [27]. Therefore, the co-emergence of *bla*_OXA−1_, *catB3*, and *arr-3* genes in MDR ESBL-producing *E. coli* strains in dairy environments may significantly threaten human and animal health. Thus, in the present study, we investigated the co-emergence and whole genome sequence-based characterization of *bla*_OXA−1_, *catB3*, and *arr-3* genes in MDR and ESBL-producing *E. coli* strains. In addition, the *in-vitro* HGT potential and efficiency were evaluated by conducting mating experiments. Multiple bioinformatic tools were used for comprehensive analysis of whole genome sequence data including molecular typing, phylogenetic analysis, identification of acquired ARGs, plasmid replicon types (PRTs), mobile genetic elements (MGEs), genetic environment analysis of *bla*_OXA−1_, *catB3*, and *arr-3* genes, prophages, and genomic Islands (GIs).

## 2. Materials and Methods

### 2.1. Bacterial Isolation and Identification of ESBL Strains

A total of 209 samples from different sources, including feces, slurry, soil, water, and milk, were collected from a dairy farm in Xinjiang province, China, whose details are given in the earlier published version [29]. First, the samples were cultured in tryptic soya broth for enrichment purposes, then streaked on MacConkey agar for selection and differentiation of *E. coli*. One or more isolated lactose fermenting colonies appearing red or pink in color on MacConkey agar were sub-cultured on Eosin Methylene Blue agar for further purification and differentiation. The purified colonies were stored in 30% glycerol at −80°C before further processing. The species identification was carried out by automatic bacterial identification systems with the VITEK-2 system following the manufacturer's guidelines (BioMerieux, France) [30] and matrix-assisted laser desorption ionization-time of flight (MALDI-TOF) as previously determined [20]. The ESBL strains were identified by standard broth microdilution assay according to the recommendations of the Clinical Laboratory and Standard Institute [31]. One isolated colony was inoculated in Mueller Hinton (MH) broth and incubated at 37°C overnight. Then, the culture was adjusted to 0.5 McFarland standard by measuring the optical density (OD) with a spectrophotometer. To each well of a 96-well microtitration plate, 100 μL of fresh MH broth was added, then 50 μL of adjusted inoculum and antibiotic working solutions (cefotaxime alone; 0.25–64 μg/mL and in combination with clavulanate; 0.25/4−64/4 μg/mL each in separate wells and same plate) was added from 1^st^−11^th^ well and 1^st^−10^th^ well respectively for all strains while keeping the 11^th^ and 12^th^ well as positive and negative control, respectively. The plate was incubated at 37°C for 16–20 h. The OD of the plate was recorded before and after incubation and interpreted accordingly to identify ESBL strains.

### 2.2. Identification of *bla*_OXA−1_, *catB3*, and *arr-3* Genes

The ESBL-producing strains were further subjected to antimicrobial susceptibility against different antibiotics. It was identified that seven *E. coli* strains showed phenotypic resistance to three classes of antibiotics, including RIF (200 μg/mL), chloramphenicol (≥32 μg/mL), and ampicillin (AMP) (≥32 μg/mL) by agar dilution assay. The DNA of phenotypically resistant strains was extracted using a TIANamp bacteria DNA kit (DP302, TIANGEN, China) and subsequently screened for *bla*_OXA−1_, *catB3*, and *arr-3* genes by PCR using the primers and conditions described previously [32–34]. The positive isolates were stored in 30% glycerol at −80°C for further processing.

### 2.3. Antimicrobial Susceptibility Testing and Multidrug Resistance Index (MDRI) Calculation

The minimum inhibitory concentrations (MICs) of 15 antibiotics (AMP, cefotaxime (CTX), MEM, ciprofloxacin (CIP), amikacin, gentamicin, tetracycline, tigecycline, trimethoprim–sulfamethoxazole (SXT), florfenicol, colistin sulfate (CS), fosfomycin, RIF, and chloramphenicol) was determined by broth microdilution assay for all strains harboring *bla*_OXA−1_, *catB3*, and *arr-3* genes. The results were interpreted using the guidelines of the Clinical Laboratory and Standard Institute (CLSI, 2020: M100) and the European Committee on Antimicrobial Susceptibility Testing clinical breakpoints (EUCAST, v.12.0) (http://www.eucast.org/clinical_breakpoints/). *E. coli* ATCC 25922 was used as a quality control strain in this study. The MDRI is the degree of antibiotic resistance from bacterial strains to overall tested antibiotics. The MDRI value ≤0.2 indicates the seldom use of antibiotics in a particular environment, while > 0.2 indicates a high risk of antibiotic exposure [35, 36]. The MDRI can be calculated by a simple formula given below:

(1) $$\begin{array}{c} \text{MDRI}=\frac{a}{b}, \end{array}$$

where *a* indicates the number of antibiotics against which a bacterial strain exhibited resistance phenotypically, and *b* is the total number of tested antibiotics [35, 36].

### 2.4. Mating Experiment

The mating experiment examined the transferability of the *bla*_OXA−1_, *catB3*, and *arr-3* genes. For this purpose, *E. coli* positive strains three genes were used as the donor strains, and *E. coli* J53 (sodium azide resistance) was employed as the recipient strain [20]. Briefly, fresh cultures of donor and recipient strains in LB broth were adjusted to 0.5 McFarland standard and mixed with a 1:1 ratio. The mixed culture was incubated overnight at 37°C, and 100 μL of the culture was plated onto LB agar plates containing RIF (200 μg/mL) and sodium azide (200 μg/mL) for selecting *arr-3* harboring transconjugants, chloramphenicol (32 μg/mL) and sodium azide (200 μg/mL) for *catB3*-positive transconjugants, and AMP (32 μg/mL) and sodium azide (200 μg/mL) for *bla*_OXA−1_-positive transconjugants. PCR and the corresponding resistance phenotypes were employed to verify the transfer of *bla*_OXA−1_, *catB3*, and *arr-3* genes in transconjugants. The frequency of transconjugation was determined by the colony count of transconjugants per recipient.

### 2.5. Whole Genome Sequencing (WGS) and Pangenome Analysis

Bacterial isolates with resistance phenotypes and co-harboring *bla*_OXA−1_, *catB3*, and *arr-3* genes were selected for WGS. Briefly, each isolate was cultured in LB broth containing the respective antibiotics and incubated overnight at 37°C with vigorous shaking at 160 rpm. The genomic DNA was extracted using a TIANamp Bacteria kit (DP302, TIANGEN, China). The purity and concentration of DNA were analyzed using a NanoDrop OneC microUV-Vis Spectrophotometer (Thermo Fisher Scientific) and further by gel electrophoresis. The sequencing was carried out by Illumina HiSeq 2500 (Novogene, Guangzhou, China) to acquire the short-read data. FastQC checked the quality of the sequence data [37]. The pangenome analysis was carried out using various bioinformatics tools such as GeneMarkS to retrieve CDS [38], tRNAscan-SE to predict tRNA genes [39], rRNAmmer for rRNA genes [40], Rfam database to predict small nuclear RNAs [41], IslandPath-DIOMB program to identify GIs [42], PHAST for prophage prediction [43], and CRISPRFinder for CRISPR identification [44].

### 2.6. Bioinformatics Analysis

The datasets acquired from Illumina HiSeq 2500 were assembled *de novo* into contigs using SPAdes while the draft genome data was annotated using the online server, Rapid Annotation Subsystems Technology (https://rast.nmpdr.org/rast.cgi) [45, 46]. The acquired antibiotic resistance genes (AARGs), PRTs, and MGEs were identified by ResFinder 4.1 (https://cge.food.dtu.dk/services/ResFinder/) by selecting AARGs and *E. coli* as target species, with 95% ID threshold and 80% minimum length [47], PlasmidFinder 2.1 (https://cge.food.dtu.dk/services/PlasmidFinder/) using *Enterobacteriaceae* database with 95% identity and 85% query coverage [48], and MobileElementFinder v 1.0.3 (https://cge.food.dtu.dk/services/MobileElementFinder/) with minimum sequence identity of ≥90% and alignment coverage ≥85% using CGE server (https://cge.food.dtu.dk/services/), respectively. Thereafter, the molecular typing, including multilocus sequence typing (MLST), core genome MLST, phylogenetic grouping, and phylogenetic tree, was determined using MLST 2.0 [47], cgMLSTFinder 1.2, Clermontyping, and PHYLOViZ [48], tools respectively. The phylogenetic tree was visualized in Interactive Tree of Life (iTOL) (iTOL: Upload a new tree (embl.de)) using the Newick tree format [49]. The BRIG-0.95 and EasyFig 2.2.5. tools were used to generate the circular and linear genome comparison, respectively, between *E. coli* strains of this study and relevant genome sequences from the NCBI database [50, 51]. The GraphPad Prism version 8.2.1 was used to construct the bar graph and heat maps to view the distribution of ARGs, PRTs, MGEs, and other traits.

### 2.7. Data Availability

The complete genome sequences of all isolates have been deposited in the NCBI database under the BioProject ID: PRJNA1049405.

## 3. Results and Discussion

### 3.1. Identification and Resistance Phenotypes of *E. coli* Strains Harboring *bla*_OXA−1_, *catB3*, and *arr-3* Retrieved From the Dairy Farm Environment

Seven bla_OXA−1_, catB3, and arr−3 co-harboring *E. coli* strains were identified from 46 MDR *E. coli* strains recovered from the selected dairy farm environment in Xinjiang, China [29]. According to our knowledge, this can be the first study reporting the co-occurrence of bla_OXA−1_, catB3, and arr-3 in *E. coli* strains isolated from the dairy farm environment. The previous studies conducted by different researchers identified the catB3 harboring *E. coli* strains in diverse habitats and geographic regions, such as chronic wounds of patients in Ghana [52], avian *E. coli* [53], and uropathogenic *E. coli* isolated from pediatrics in India [54], and bloodstream infections of a patient admitted in a Chinese hospital [55]. Similarly, bla_OXA−1_-carrying *E. coli* strains have been reported in various settings from China [56], Nigeria [57], Belgium [58], and other countries [59–61]. Not all bla_OXA−1_ gene variants are identified as ESBL-producing. However, few variants can be evolved to produce ESBL and carbapenemase, which makes the occurrence of this gene worrying and can add more to the AMR crises [62]. The arr-3-carrying *E. coli* strains have also been reported recently [34, 52, 63–65]. Co-occurrence of bla_OXA−1_-catB3-arr-3 has been reported previously in *Proteus mirabilis* and Salmonella species [66]. Although another study reported the aac (6′)-lb-cr—bla_OXA−1_-catB3 cluster in *E. coli* ST410 isolated from a natural water environment [67], here we identified the *E. coli* strains co-harboring bla_OXA−1_-catB3-arr-3 from the dairy farm environment. Antimicrobial susceptibility testing showed that all of the seven strains were resistant to AMP, CTX, RIF, chloramphenicol (C), and SXT. At the same time, *n* = 6 strains were resistant to CIP and tetracycline (TET), and *n* = 5 were resistant to florfenicol (FFC) with varying ranges of MICs (Tables 1 and S1). In addition, strains also exhibited low-level resistance to amikacin (AMK, *n* = 3), gentamicin (GEN, *n* = 4), tigecycline (TIG, *n* = 1), and fosfomycin (FOS, *n* = 1). In contrast, none of these strains exhibited resistance to MEM and CS (Table S1). All of the strains were identified as MDR (nonsusceptible to at least one antimicrobial agent from three or more antimicrobial classes) (Table S2) and ESBLs (nonsusceptible to CTX alone and in combination with clavulanate). Recent reports have been on ESBL-producing MDR *E. coli* from different countries [56–68]. The MDRI index value of studied isolates was 0.57–0.71 (Table 1), which may indicate the harmful exposure of the particular environment to antibiotic contamination. This is consistent with the findings from earlier studies conducted in different environmental settings [10, 69]. Moreover, all strains were identified as ESBL, and ESBL-producing strains are mostly recognized as MDR, evidenced by increased MDRI in this study, and present a great concern to animal and human health [29, 70].

### 3.2. Pangenome Analysis and Comparison of *bla*_OXA−1_, *catB3*, and *arr-3* Positive *E. coli* Strains Isolated From Dairy Farm Environment in China

**Figure 1:**
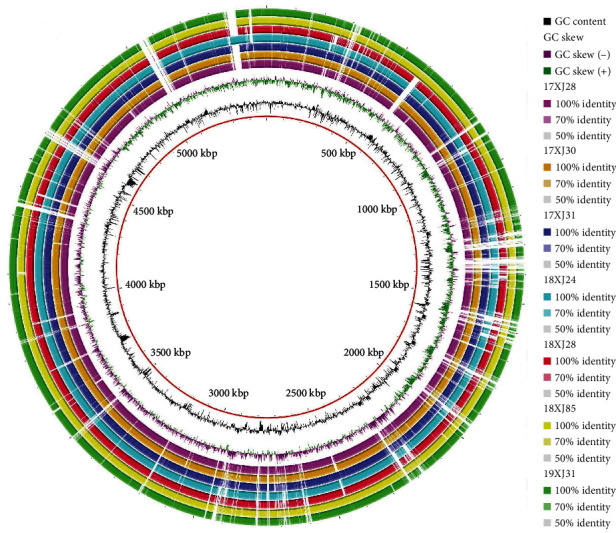
Circular comparison of the whole genome sequence of the seven *E. coli* strains carrying *bla*_OXA−1_, *catB3*, and *arr-3* genes.

The whole genome sequence data of the seven *E. coli* strains were analyzed using various bioinformatics tools. The genome sizes of the *E. coli* strains co-harboring *bla*_OXA−1_, *catB3*, and *arr-3* ranged from 5.3 to 7.0 Mb with GC content of 50.58% to 51.55%. The number of annotated genes ranged from 5114 to 5815, CRISPR segments from 9 to 90, GIs from 1 to 15, and prophages from 9 to 126 (Table S3). The circular comparison of the whole genome of the seven *E. coli* strains shared a more remarkable similarity, as shown in Figure 1.

### 3.3. Molecular Typing of *bla*_OXA−1_, *catB3*, and *arr-3* Positive *E. coli* Strains

**Figure 2:**
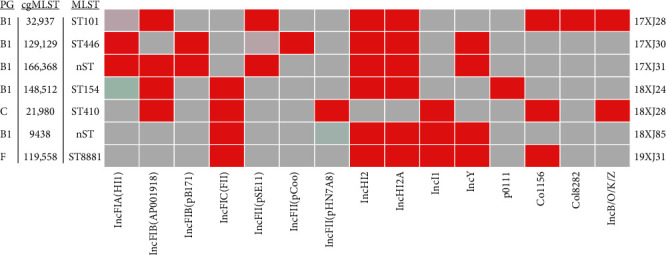
Molecular typing of the seven *E. coli* strains co-harboring *bla*_OXA−1_, *catB3*, and *arr-3* genes. The heatmap shows the identified plasmid replicon types (red rectangles) along with multilocus sequence typing (MLST), core genome MLST (cgMLST), and phylogeny group (PG) on the left panel.

The molecular typing of the *E. coli* strains was determined by using various typing tools such as MLST 2.0, cgMLSTFinder 1.2, PlasmidFinder 2.1, and CSIPhylogeny using the CGE server (https://genomicepidemiology.org/services/). MLST revealed that five of the seven isolates belonged to five distinct sequence types (STs), including ST101, ST446, ST410, ST154, and ST8881, while the other two were grouped under novel or new STs (Figure 2). Among the STs, ST154 clones have recently been reported from Portugal [71], Germany [72], and Pakistan [73]. The *E. coli* clones of ST101 were declared as global epidemic *E. coli* clones associated with acquiring ESBLs and other resistance genes [19, 74–76]. *E. coli* ST446 clone has also been reported worldwide from various countries, including China [77–79]. Previously, ST410 *E. coli* has been reported from swine-origin in the USA [80], urinary tract of patients in Egypt carrying the *bla*_OXA−1_ gene and diverse plasmid replicons [81], and humans and environment in Southeast Asia [79], and has also recognized as an emerging high-risk clone internationally [82–84]. Results from this study represent the first report of ST410 *E. coli* from the dairy environment, suggesting the potential of dairy as a source for ST410. Previously, ST8881 was the least reported *E. coli* lineages, such as in pediatric patients in Qatar and other human populations from Tanzania, the USA, the UK, Australia, Spain, and Bangladesh. We first reported the ST8881 *E. coli* lineage from China isolated from a dairy farm environment. The distinct STs among the seven strains indicate the genetic diversity of *bla*_OXA−1_, *catB3*, and *arr-3* positive *E. coli*. Additionally, we conducted cgMLST analysis based on the total number of allelic loci (2513) to the number of alleles called in the core genome database (Table S4). The percent alleles identity was set at ≥90%, and it was identified that 4/7 of the strains, 17XJ28, 17XJ30, 18XJ28, and 19XJ31, fulfilled this criterion and revealed distinct cgSTs, 32,937, 129,129, 21,980, and 119,558, respectively. The percent allele identity of 17XJ31, 18XJ24, and 18XJ85 was noted to be 55.51%, 89.61%, and 49.14%, which belong to cgMLST 166,368, 148,512, and 9438, respectively (Figure 2).

The plasmid replicon typing of seven *E. coli* strains was done using the PlasmidFinder 2.1 tool under CGE (https://cge.food.dtu.dk/services/PlasmidFinder/). The analysis revealed 15 PRTs acquired by the seven *E. coli* strains, including IncFIA (HI1) (*n* = 2), IncFIB (AP001918) (*n* = 4), IncFIB (pB171) (*n* = 2), IncFIC (FII) (*n* = 4), IncFII (pHN7A8) (*n* = 1), IncFII (pCoo) (*n* = 1), IncFII (pSE11) (*n* = 2), IncHI2 (*n* = 6), IncHI2A (*n* = 6), IncI1-I (Alpha) (*n* = 3), IncY (*n* = 4), p0111 (*n* = 1), Col156 (*n* = 3), Col8282 (*n* = 1), and IncB/O/K/Z (*n* = 2) (Figure 2). Among different PRTs, IncF plasmids were acquired more frequently than others and carried much of the antibiotic resistance and virulence genes. IncHI2 and IncHI2A plasmids were found to be acquired by all seven *E. coli* strains except 18XJ28. IncFII (pCoo), IncFII (pHN7A8), p0111, and Col8282 plasmids were noted to be acquired by individual *E. coli* strains, 17XJ30, 18XJ28, 18XJ24, and 17XJ28, respectively. Moreover, IncFIA (HI1)/IncFIB (pB171)/IncFII (pSE11)/IncB/O/K/Z, Col156/IncI1-I (Alpha), and IncFIB (AP001918)/IncFIC (FII)/IncY plasmids were acquired by 2, 3, and 4 *E. coli* strains, respectively (Figure 2). Previously, *E. coli* bearing IncFIA (HI1), IncFIB (AP001918), IncFIB (pB171), IncFIC (FII), IncFII (pHN7A8), IncFII (pCoo), IncFII (pSE11), IncHI2, IncHI2A, IncI1, IncY, p0111, Col156, Col8282, and IncB/O/K/Z plasmids were reported from animal, human and environment [10, 14, 54, 85]. The presence of diverse types of plasmid replicons may harbor diverse ARGs and might increase the possibility of the spread of ARGs through conjugation. The phylogenetic grouping of the seven *E. coli* strains was determined by *in-silico* Clermont typing, and the majority of strains belonged to group B1 (5/7, 71.4%), followed by C (1/7, 14.3%) and F (1/7, 14.3%) (Figure 2).

### 3.4. Phylogenetic Relationship of *bla*_OXA−1_, *catB3*, and *arr-3* Gene-Positive *E. coli* Strains Isolated From China Dairy Farm Environment with Other Strains From NCBI

**Figure 3:**
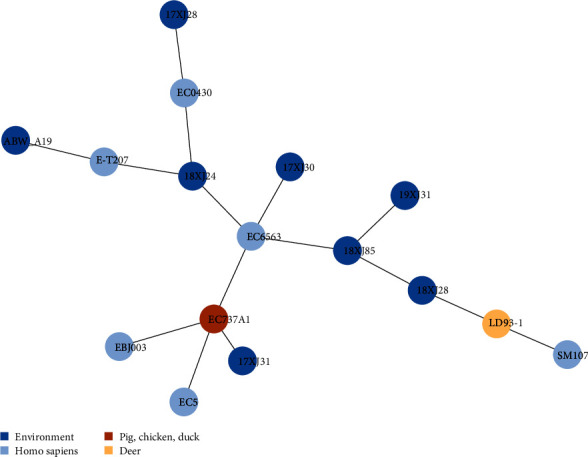
The comparative phylogenetic tree of seven *E. coli* strains from this study and nine strains (EC0430, EC6563, E-T207, ABW_A19, EC5, EBJ003, EC737A1, LD93-1, and SM107) co-harboring *bla*_OXA−1_, *catB3*, and *arr-3* genes were retrieved from the NCBI database.

The phylogenic relationship of the seven *E. coli* strains co-harboring *bla*_OXA−1_, *catB3*, and *arr-3* genes of the present study and other strains retrieved from the NCBI database was determined by phylogeny construction by PHYLOViZ online based on pubMLST allelic profile. The distance matrix of allelic profiles is given in Table S5. The comparative phylogenetic analysis clustered 17XJ28 and 17XJ30 with human, EC0430 (accession no: CP123046.1) and EC6563 (accession no: CP095856.1) *E. coli* strains isolated from sputum and urine samples, respectively, in China. Similarly, the 18XJ24 strain was identified as most closely related to human (E-T207, accession no: CP090277.1) and environmental *E. coli* strain (ABW_A19, accession no: CP067307.1) isolated from ICU patient and wastewater in China and Switzerland, respectively (Figure 3). Moreover, 17XJ31 was more closely related to two human strains, EC5, accession no: CP060969.1, and EBJ003, accession no: CP086334.1 isolated from wound and urine samples, respectively, and a food animal strain (EC737A1, accession no: CP100005.1) recovered from the chicken gut in China. The other three strains of the present study, 18XJ28, 18XJ85, and 19XJ31, showed closed genetic relatedness with LD93-1 (accession no: CP047662.1) and SM107 (accession no: CP130667.1) strains isolated from feces and urine samples of deer and human, respectively in China. This may indicate that the *E. coli* strains co-harboring *bla*_OXA−1_, *catB3*, and *arr-3* genes are circulating between human, animal, and environmental settings.

### 3.5. Analysis of Transferability of the *bla*_OXA−1_, *catB3*, and *arr-3* Genes Into *E. coli* J53

To investigate the transferability of *bla*_OXA−1_, *catB3*, and *arr-3* genes, all seven strains were subjected to a mating experiment using *E. coli* J53 (sodium azide resistance) as the recipient strain. *E. coli* C600 (rifampicin resistance) was not used as the recipient because all donor strains were resistant to rifampicin due to the presence of *arr-3*. Because of the co-presence of *bla*_OXA−1_, *catB3*, and *arr-3* in the donor strains, phenotypes for rifampicin, chloramphenicol, and beta-lactam resistance were successfully transferred to *E. coli* J53. This may be evidenced by a previous study conducted by Wang et al. [66], which reported the possible *in-vitro* horizontal transfer of these genes from *P. mirabilis* into *E. coli*. These findings may indicate that these genes were located on conjugative plasmids and transferred horizontally to trans-conjugants. Previously, multiple studies reported the location of these genes on IncF plasmids [86–88]. The conjugation efficiency of *catB3*-positive strains was noted from 2.65 ± 0.5 × 10^−3^ to 3.77 ± 0.8 × 10^−1^ trans-conjugants/recipient (Table 2). In comparison, the transfer frequency of *arr-3* was lower than *catB3* but higher than *bla*_OXA−1_ 1.60 ± 0.7 × 10^−3^ to 3.10 ± 0.3 × 10^−1^ trans-conjugants per recipient (Table 2). The conjugation frequency of *bla*_OXA−1_ ranged from 1.45 ± 0.5 × 10^−3^ to 4.90 ± 0.6 × 10^−2^ per recipient. However, the co-conjugation frequency of *arr-3*, *catB3*, and *bla*_OXA−1_ in all strains was much lower, ranging from 2.90 ± 0.5 × 10^−5^ to 3.85 ± 0.3 × 10^−3^ per recipient (Table 2). The difference in the conjugation frequency may be due to the addition of different antibiotics, and these antibiotics act as selective drivers for conjugation dynamics. Lopatkin et al. [89] demonstrated that conjugation dynamics are regulated by antibiotic-mediated selection, which can promote or suppress the conjugation frequency. These findings indicate that conjugation-mediated HGT can disseminate these three ARGs.

### 3.6. Genetic Environment of *bla*_OXA−1_, *catB3*, and *arr-3* Among the Isolated *E. coli* From Dairy Farm Environment in China

**Figure 4:**
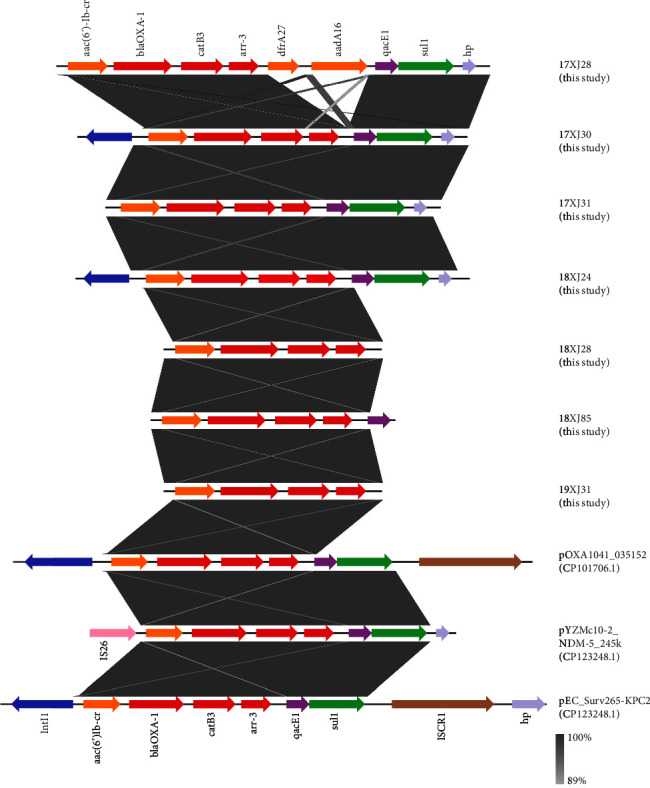
The linear genetic environment of the *bla*_OXA−1_, *catB3*, and *arr-3* genes in the seven *E. coli* strains highlighted in red color and comparison with NCBI retrieved isolates.

To understand the mechanism of HGT, it is necessary to characterize the ARGs' genetic contexts (Figure 4). The genome contigs carrying *bla*_OXA−1_, *catB3*, and *arr-3* genes in all of the seven *E. coli* strains were blasted with three *E. coli* plasmids retrieved from NCBI database: pOXA1041_035152 (accession no: CP101706.1) recovered from the blood of human in Sichuan, China; pYZMc10-2_NDM-5_245k (accession no: CP123248.1) isolated from feces of chicken in Yangzhou, China; and pEC_Surv265-KPC2 (Accession No: CP104953.1) retrieved from bronchial secretions of a patient in Giessen, Germany. The genetic backbone of the three plasmids was found to be highly identical to the backbone of the present study strains, which were predominantly carrying *aac* (*6′*)-*Ib-cr*-*bla*_OXA−1_-*catB3*-*arr-3*-*qacE*-*sul1* genetic backbone in their genetic context (Figure 4). The plasmids, pOXA1041_035152 and pEC_Surv265-KPC2 genetic context showed that *aac* (*6′*)-*Ib-cr*- *bla*_OXA−1_-*catB3*-*arr-3*-*qacE*-*sul1* gene array was flanked by *IntI1* integron in the upstream and *ISCR1* mobile element in downstream which may play an essential role in the horizontal spread of this gene array. The presence of *IntI1* integron in the upstream of *bla*_OXA−1_-*catB3*-*arr-3 genes* in *E. coli* 17XJ30 and 18XJ24 may indicate their location on conjugative plasmids and can mediate the horizontal transfer of this gene array. In the 17XJ28 strain, *dfrA27* and *aadA16* genes conferring resistance to trimethoprim and aminoglycosides were also found to be located within the same contig. However, the contigs of 18XJ28, 18XJ85, and 19XJ31 were missing some of the genetic structures due to the small contigs' length. A similar genetic backbone was also previously reported in *P. mirabilis* and *Salmonella* species [66]. The plasmid pYZMc10-2_NDM-5_245k was carrying an IS26 mobile element upstream of this gene array, which replaced the *intI1* integron. Previous studies have discussed the significant role of the IS26 mobile element in the spread of multiple ARGs, especially in environmental settings [90, 91]. IS26 is mainly associated with class 1 integrons, and its multiple insertions in genomes can lead to the rearrangement of resistance loci [92]. A previous study in India reported the IS26-mediated translocation of *aac* (*6′*)-*Ib-cr*, *bla*_OXA−1_, and *catB3* genes in the *K. pneumoniae* strain [93].

### 3.7. AARGs Among Seven MDR *E. coli* Strains Isolated From Dairy Farm Environment in China

**Figure 5:**
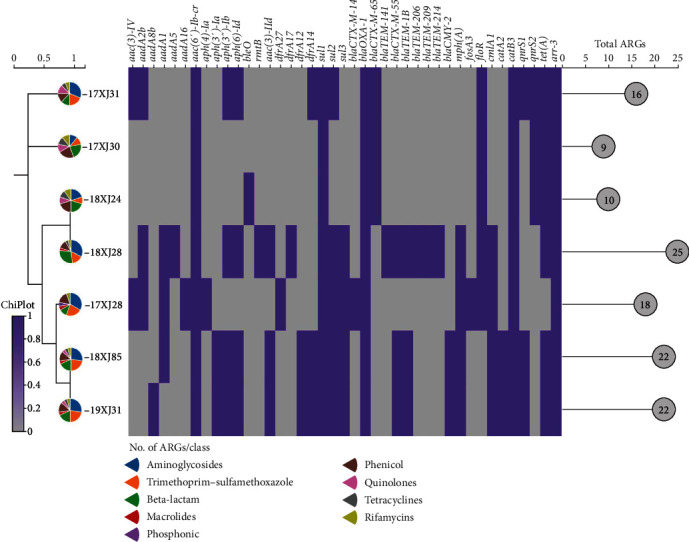
The distribution of AARGs in the seven *E. coli* strains identified in this study. The purple box indicates the presence of genes, while the light gray box indicates the absence. The pie chart on the tree branches indicates the number of AARGs harbored by each isolate from each antimicrobial class. Moreover, the lollipop mark at the base indicates the total number of ARGs acquired by each *E. coli* strain.

The prevalence of other AARGs identified by ResFinder using the CGE server in the seven *E. coli* strains revealed a significant variation in the number and frequency of distribution between and within the antibiotic category. The total number of AARGs among the seven *E. coli* strains is 122, with an average of 17.4 (122/7) per strain. 18XJ28 strain harbored 25 AARGs, the highest number among the seven strains. Along with the co-occurrence of *bla*_OXA−1_, *catB3*, and *arr-3* genes, 38 other AARG variants were identified in the seven *E. coli* strains, conferring resistance to multiple antibiotic classes such as quinolones, sulfonamides, trimethoprim, aminoglycosides, tetracyclines, macrolides, florfenicol, and *β*-lactams (Figure 5). Furthermore, the highest number of the AARGs belonged to aminoglycosides (*n* = 14/41, 34.2%), followed by *β*-lactams (*n* = 10/41, 24.4%), trimethoprim (*n* = 4/41, 9.8%), sulfonamides, (*n* = 3/41, 7.3%), chloramphenicol (*n* = 3/41, 7.3%), florfenicol (*n* = 2/41, 4.9%), quinolones (*n* = 2/41, 4.9%), macrolides (*n* = 1/41, 2.4%), tetracyclines (*n* = 1/41, 2.4%), and rifamycin's (*n* = 1/41, 2.4%). Among the acquired ARGs, the most widespread resistance genes were *aac* (*6′*)-*Ib-cr* and *sul1*, conferring resistance to aminoglycosides and sulfonamides, respectively (*n* = 7/7, 100% of the *E. coli* isolates). In addition to *aac* (*6*′)-*Ib-cr* and *sul1*, other ARGs responsible for aminoglycosides resistance included *aph* (*3*″)-*Ib*, *aph* (*6*)-*Id* (*n* = 4/7 each), *aadA2b*, *aadA1* (*n* = 3/7), *aac* (*3*)-*IV*, *aac* (*3*)-*IId*, *aph* (*3*′)-*Ia* (*n* = 2/7), *aadA8b*, *aadA5*, *aph4-Ia*, *bleO*, *rmtB*, and *aadA16* (*n* = 1/7 each), and sulfonamides resistance genes *sul2* (*n* = 5/7) and *sul3* (*n* = 4/7). The distribution of 11 ARGs conferring resistance to *β*-lactams were as follows: *bla*_CTX-M−14_, *bla*_TEM−141_, *bla*_TEM−206_, *bla*_TEM−209_, and *bla*_TEM−214_ (*n* = 1 each), and *bla*_CTX-M−65_, *bla*_CTX-M−55_, and *bla*_TEM−1B_ (*n* = 3). Additionally, four trimethoprim genes, *dfrA27* (*n* = 1), *dfrA17* (*n* = 1), *dfrA12* (*n* = 2), and *dfrA14* (*n* = 3), and two chloramphenicol genes apart from *catB3*, *cmlA1* (*n* = 4) and *catA2* (*n* = 2) were also identified. Moreover, one fosfomycin (*fosA3*, *n* = 1), one florfenicol (*floR*, *n* = 5), and two quinolone genes (*qnrS1* and *qnrS2*; *n* = 3 each) were also identified. The macrolide resistance gene (*mphA*) and tetracycline gene (*tet* (*A*)) were detected in *n* = 4 and *n* = 6 strains, respectively. *E. coli* strains carrying a diverse range of AARGs have been reported previously from various environmental settings such as aquaculture in Portugal [94], cryoconite and ice glaciers in Poland [9], migratory water birds in Spain [95], drinking water system in China [96], wastewater treatment plant in Brazil [97], and freshwater lakes in India [10]. As far as we know, very few studies have reported the diversity of AARGs acquired by *E. coli* strains from dairy farm environments. The co-occurrence of *bla*_OXA−1_, *catB3*, and *arr-3*, along with a diverse range of AARGs conferring resistance to antibiotics of multiple classes, indicate the potential of the dairy production environment as a reservoir for MDR bacteria.

### 3.8. MGEs Among Seven MDR *E. coli* Strains Isolated From Dairy Farm Environment in China

**Figure 6:**
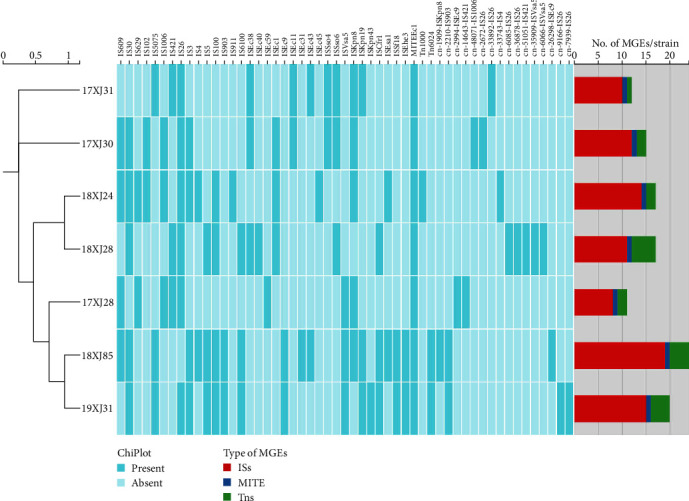
The mobile genetic elements (MGEs) acquired by seven *E. coli* strains; the dark-colored rectangles indicate the presence, while the light color indicates the absence of a particular genetic trait. The bar graph at the base shows the MGEs acquired by each strain, which includes ISs (insertion sequences), MITE (miniature inverted repeat elements), and Tns (transposons).

MGEs, such as insertion sequences (ISs) and transposons (Tns), are widely present in the bacteria genome and play an important role in the remodeling of the host genome [98]. They can transfer multiple genes and larger DNA regions into the genetic makeup, including different genome replicons such as plasmids, viral genomes, and bacterial chromosomes [99]. In the present study, we identified MGEs using the IS finder database (ISfinder (biotoul.fr)) using the CGE server. The total number of MGEs harbored by the seven strains varied from 123 to 222 regardless of sequence similarity and coverage (0%–100%). The distribution of MGEs, such as small MGEs, MGEs carrying genes (Tns), and conjugative MGEs, are summarized in Table S6. Among the MGEs, small MGEs, especially the ISs, were found to be most abundant, followed by gene-carrying MGEs (Tns) and conjugative MGEs in all seven *E. coli* strains. All *E. coli* strains were carried at least one conjugative MGE except 18XJ24 (Table S6). Then, the MGEs were filtered with a minimum sequence identity of ≥90% and alignment coverage of ≥85%, listed in Figure 6. A total of 53 MGEs that met the criteria were identified, and it was noted that 18XJ85 harbored the highest number of MGEs (*n* = 24), followed by 19XJ31 (*n* = 20), 18XJ24 and 18XJ28 (*n* = 17 each), 17XJ30 (*n* = 15), 17XJ31 (*n* = 12), and 17XJ28 (*n* = 11). Among the identified MGEs, ISs were the most abundant (*n* = 34/53, 64.2%), followed by composite transposons (CTs) (*n* = 16/53, 30.2%), unit transposons (UTs) (*n* = 2/53, 3.7%), and miniature inverted-repeat transposable elements (MITEs) (*n* = 1/53, 1.9%) (Figure 6). Moreover, none of the strains carried other MGEs such as MICs, conjugative integrative mobile elements, integrative mobile elements, and integrative conjugative elements under this criterion (minimum sequence identity of ≥90% and alignment coverage ≥85%). Among the ISs, IS26 was found to be the most abundant (85.7%, *n* = 6/7), followed by IS30 and ISKpn8 (71.4%, *n* = 5/7 each), IS609, IS3, and IS100 (57.1%, *n* = 4/7 each), IS5075, IS1006, IS421, IS5, IS6100, ISEc38, ISEc1, ISSso6, ISVsa5, ISKpn19, and ISCfr1 (42.8%, *n* = 3/7), IS629, IS102, IS4, IS903, ISEc9, ISEc11, ISEc43, ISSso4, ISEsa1, ISSfl8, and ISEhe3 (28.6%, *n* = 2/7) while IS911, ISEc40, ISEc59, ISEc31, ISEc45, and ISKpn43 were rare and occurred in a single strain. It was noted that most of the *E. coli* strains were equipped with two and three different types of ISs. The predominance of IS26 in environmental *E. coli* strains has also been noted previously [90, 91, 94]. The presence of IS30 as a new IS was first reported by Caspers et al. [100] in the *E. coli* K12 strain and recently in environmental strains [101, 102]. However, MITEEc1 was acquired by all the *E. coli* strains and was noted to be the most predominant among MITEs (100%), which follows the findings of a previous study [94]. The gene carrying MGEs (Tns), Tn1000 unit transposon, was identified in only 18XJ24 and Tn6024 in 18XJ85 and 19XJ31 strains, which were also reported previously [103, 104]. All strains harbored at least one composite transposon (Figure 6), which facilitates the spread of ARGs through transposition [103]. In this study, we have extensively discussed the large number of MEGs that can be the potential mediator of horizontal transfer of ARGs within and between the bacterial species in the environment and ultimately to human and animal settings through various routes.

### 3.9. ARGs, MGEs, and PRTs Associated with Prophages and GIs in Seven MDR *E. coli* Harboring *bla*_OXA−1_, *catB3*, and *arr-3* Genes Isolated From Dairy Farm Environment in China

Prophages frequently participate in host survival mechanisms and assist the host genome to become genetically more diverse. Prophages also allow diverse genes to spread horizontally [105]. The number of prophages and GIs in this study varied among the seven *E. coli* strains (described in Table S3). The prophage genome segments of *E. coli* strains 17XJ28, 17XJ31, 18XJ28, and 19XJ31 harbored ARGs. The role of prophages in harboring various ARGs has also been studied previously in different ecological niches [105–107]. The prophage segments in 17XJ28 harbored *aac* (*6′*)-*Ib-cr*, *aadA16*, *dfrA27*, *sul1*, *bla*_OXA−1_, *arr-3*, *catB3*, and *mph* (*A*) genes which confer resistance to aminoglycosides, trimethoprim, sulfonamides, beta-lactams, rifampicin, chloramphenicol, and macrolides. The 17XJ28 prophage segment also carried an IncFIB (AP001918) plasmid type. Recently, Pfeifer and Rocha [108] also described new elements known as prophage-plasmids or phage-plasmids. These can be transferred between the cells horizontally as viruses and vertically within the cell lineages as plasmids. These elements can promote recombination events, including mobile elements, defense systems, and antibiotic-resistance genes. Moreover, the prophage genomic component of 17XJ31 harbored genes confer resistance to quinolones (*qnrS1* and *qnrS2*) and tetracycline (*tetA*) along with IncY PRT and ISKpn19 MEG (Table 3). The *aph* (*3′'*)-*Ib* and *sul2* genes were identified in the prophage genome 18XJ28 and the *dfrA12* gene in 19XJ31. The prophage genomes of all *E. coli* strains carried at least one mobile element except the 19XJ31 strain (Table 3). These findings highlight that prophages may act as reservoirs or gene carriers for ARGs along with mobile elements and their spread by HGT, evidenced by a previous study [106]. The GIs are the gene clusters within a genome ranging in size from a few to 500 kb and were first defined by Hacker et al. [109]. The sequence analysis of GIs identified in the present study showed that only one strain (17XJ30) harbored the *tet* (*A*) gene along with IncFIA (HI1) PRT and IS30 mobile element, while the other three strains, 18XJ24, 18XJ28, and 19XJ31 were only carrying the mobile elements (Table 3). However, the GIs identified in 17XJ28, 17XJ31, and 18XJ85 did not harbor ARGs, PRTs, or MGEs. GIs can disseminate between bacterial species through conjugation, transformation, and transduction [110]. Still, conjugation is considered a primary dissemination mode [111]. GIs can also serve as reservoirs, procure gene elements, and may be disseminated through HGT [111].

## 4. Conclusion

This study reported the co-emergence of *bla*_OXA−1_, *catB3*, and *arr-3* in *E. coli* strains isolated from the dairy farm environment in China. Our results revealed multidrug resistance phenotypes, a large pool of acquired ARGs, and their horizontal spread by diverse PRTs and MEGs. The genetic environment of *bla*_OXA−1_, *catB3*, and *arr-3* highlighted the ability to procure the novel genetic backbone, which may participate in its increased persistence in the environment and then spread between and within bacterial species. *E. coli* colonizes the gut of humans and animals, which may transfer acquired ARGs to other members of intestinal microbiota. The present study provided detailed information through a WGS analysis highlighting the role of the dairy farm environment in disseminating ARGs, which previously remained understudied. Therefore, this study recommends the continuous genomic-based surveillance and characterization of critical pathogenic species in the dairy production environment on a large scale in a One Health approach to identify the emergence and spread of novel resistance patterns that can suggest relevant measures and actions to limit their spread.

## Figures and Tables

**Table 1:** Antimicrobial resistance characteristics of the seven MDR *E. coli* strains carrying *bla*_OXA−1_, *catB3*, and *arr-3* genes isolated from the dairy farm environment in China.

Strain ID	Antimicrobials MIC (*µ*g/mL)														MDRI
	AMP	CTX	RIF	C	MEM	CIP	AMK	GEN	TET	TIG	SXT	FFC	CS	FOS	
17XJ28	≥512	≥64	≥300	≥256	0.5	0.5	16	≥64	16	0.5	≥128/2432	128	0.5/1	≥512	0.64
17XJ30	≥512	≥128	≥300	≥128	0.125	1	8	2	64	0.125	4/76	256	0.5	1	0.57
17XJ31	≥512	≥64	≥300	≥128	0.5	1	16	2	64	0.5	8/152	256	0.5	2	0.57
18XJ24	≥256	≥32	≥300	≥64	≤0.015	1	8	4	64	≤0.062	16/304	256	0.5	0.5	0.57
18XJ28	≥256	≥256	≥300	≥256	≤0.015	≥8	≥128	≥32	128	0.25	64/304	256	0.5	1	0.71
18XJ85	> 128	≥32	≥300	≥256	2	1	≤4	≤1	8	0.25	8/152	16	0.5	16	0.57
19XJ31	≥512	≥64	≥300	≥256	<0.062	≥16	32	256	≥512	0.125	≥128/2432	16	1	2	0.71
ATCC 25,922	8	≤0.125	4	8	≤0.125	≤0.016	4	0.25	0.5	≤0.125	≤0.5/9.5	4	0.25	≤2	—

Abbreviations: AMK, amikacin; AMP, ampicillin; C, chloramphenicol; CIP, ciprofloxacin; CS, colistin sulfate; CTX, cefotaxime; FFC, florfenicol; FOS, fosfomycin; GEN, gentamicin; MEM, meropenem; RIF, rifampicin; SXT, sulfamethoxazole-trimethoprim; TET, tetracycline; TIG, tigecycline.

**Table 2:** Conjugation characteristics of the seven *E. coli* strains carrying *bla*_OXA−1_, *catB3*, and *arr-3* genes isolated from the selected dairy farm environment in China.

Donor strain	Recipient strain	Transferability/conjugation frequency of *arr-3*	Transferability/conjugation frequency of *catB3*	Transferability/conjugation frequency of *bla*_OXA−1_	Co-transferability/conjugation frequency
17XJ28	*E. coli* J53	Transferable/(3.25 ± 0.4) × 10^−2^	Transferable/(4.30 ± 0.2) × 10^−1^	Transferable/(2.90 ± 0.2) × 10^−3^	Transferable/(2.80 ± 0.4) × 10^−4^
17XJ30	*E. coli* J53	Transferable/(4.90 ± 0.3) × 10^−1^	Transferable/(5.40 ± 0.7) × 10^−1^	Transferable/(4.25 ± 0.7) × 10^−2^	Transferable/(3.40 ± 0.7) × 10^−3^
17XJ31	*E. coli* J53	Transferable/(3.44 ± 0.1) × 10^−2^	Transferable/(2.65 ± 0.5) × 10^−3^	Transferable/(2.10 ± 0.3) × 10^−3^	Transferable/(2.90 ± 0.5) × 10^−5^
18XJ24	*E. coli* J53	Transferable/(4.35 ± 0.1) × 10^−1^	Transferable/(4.52 ± 0.3) × 10^−1^	Transferable/(4.90 ± 0.6) × 10^−2^	Transferable/(3.85 ± 0.3) × 10^−3^
18XJ28	*E. coli* J53	Transferable/(3.20 ± 0.6) × 10^−1^	Transferable/(2.75 ± 0.2) × 10^−2^	Transferable/(2.33 ± 0.4) × 10^−2^	Transferable/(2.25 ± 0.3) × 10^−4^
18XJ85	*E. coli* J53	Transferable/(1.60 ± 0.7) × 10^−3^	Transferable/(1.60 ± 0.5) × 10^−2^	Transferable/(1.45 ± 0.5) × 10^−3^	Transferable/(3.10 ± 0.1) × 10^−5^
19XJ31	*E. coli* J53	Transferable/(3.10 ± 0.3) × 10^−1^	Transferable/(3.77 ± 0.8) × 10^−1^	Transferable/(3.56 ± 0.2) × 10^−2^	Transferable/(3.63 ± 0.6) × 10^−3^

**Table 3:** ARGs, MGEs, and PRTs associated with prophages and genomic islands in seven MDR *E. coli* isolated from dairy farm environment in China.

Strains ID	Prophages			Genomic Islands		
	ARGs	PRTs	MGEs	ARGs	PRTs	MGEs
17XJ28	*aac*(*6*′)-*Ib-cr*, *aadA16*, *dfrA27*, *sul1*, *bla*_OXA−1_, *arr-3*, *catB3*, *mph*(*A*)	IncFIB(AP001918)	IS1006	None	None	None
17XJ30	None	IncY	ISEc1	*tet*(*A*)	IncFIA(HI1)	IS30
17XJ31	*qnrS1*, *qnrS2*, *tet*(*A*)	IncY	ISKpn19	None	None	None
18XJ24	None	None	ISEsa1	None	None	IS100, ISEc45, ISEsa1
18XJ28	*aph*(*3*″)-*Ib*, *sul2*	None	IS100, IS30	None	None	IS30
18XJ85	None	None	ISEsa1, ISEhe3, MITEEc1	None	None	None
19XJ31	*dfrA12*	None	None	None	None	ISSfl8

## Data Availability

Datasets used and/or analyzed during this study are available from the NCBI database under BioProject ID: PRJNA1049405 and in supporting informations.

## Supporting Information

Supporting Information The supporting information generated for this article is available in Table S1–S6. Table S1: Prevalence and phenotypes of *E. coli* strains carrying blaOXA-1, catB3, and arr-3 genes isolates from dairy farm environment in China. Table S2: Phenotypic description of MDR *E. coli* harboring blaOXA-1, catB3, and arr-3 resistance gene isolated from farm environment in China. Table S3: Genome component analysis of *E. coli* strains carrying blaOXA-1, catB3, and arr-3 genes isolates from dairy farm environment in China. Table S4: Core genome MLST (cgMLST) allelic profiles of *E. coli* strains carrying blaOXA-1, catB3, and arr-3 genes isolates from dairy farm environment in China. Table S5: Distance matrix of allelic profiles of this study and NCBI retrieved strains for phylogenetic analysis. Table S6: Mobile Genetic Elements (MGEs) identified in the core genome of blaOXA-1, catB3, and arr-3 genes carrying *E. coli* strains recovered from the dairy farm environment in China.
