# Supplementary material for: Genomic Characterization of Extended-Spectrum β-Lactamase (ESBL) Producing E. coli Harboring blaOXA−1-catB3-arr-3 Genes Isolated From Dairy Farm Environment in China
Source: Transbound Emerg Dis. 2024 Oct 11;2024:3526395. doi: 10.1155/2024/3526395 (PMC12017223; doi:10.1155/2024/3526395)
Supplement: Supporting Information — The supporting information generated for this article is available in Table S1–S6. Table S1: Prevalence and phenotypes of E. coli strains carrying blaOXA-1, catB3, and arr-3 genes isolates from dairy farm environment in China. Table S2: Phenotypic description of MDR E. coli harboring blaOXA-1, catB3, and arr-3 resistance gene isolated from farm environment in China. Table S3: Genome component analysis of E. coli strains carrying blaOXA-1, catB3, and arr-3 genes isolates from dairy farm environment in China. Table S4: Core genome MLST (cgMLST) allelic profiles of E. coli strains carrying blaOXA-1, catB3, and arr-3 genes isolates from dairy farm environment in China. Table S5: Distance matrix of allelic profiles of this study and NCBI retrieved strains for phylogenetic analysis. Table S6: Mobile Genetic Elements (MGEs) identified in the core genome of blaOXA-1, catB3, and arr-3 genes carrying E. coli strains recovered from the dairy farm environment in China. [file 3526395.f1.docx]

**Manuscript Title:** Genomic characterization of extended-spectrum β-lactamase (ESBL) producing *E. coli* harboring *bla*_OXA-1_*-catB3-arr-3* genes isolated from dairy farm environment in China

**Prevalence and phenotypes of *E. coli* strains carrying** *bla*_OXA-1_, *catB3*, and *arr-3* genes

Antimicrobial susceptibility testing showed the seven isolates were resistant to ampicillin (AMP, 100%), cefotaxime (CTX, 100%), rifampicin (RIF, 100%), chloramphenicol (C, 100%), ciprofloxacin (CIP, 85%), tetracycline (TET, 85%), trimethoprim-sulfamethoxazole (SXT, 100%), and florfenicol (FFC, 71.4%) with varying ranges of MICs (Table 1 & S1). In addition, strains exhibited low-level resistance to amikacin (AMK, 42.8%), gentamicin (GEN, 57.1%), tigecycline (TIG, 14.3%), and fosfomycin (FOS, 14.3%). In contrast, none of these strains demonstrated resistance to meropenem (MEM) and colistin sulfate (CS) (Table S1).

Table S1. Prevalence and phenotypes of *E. coli* strains carrying *bla*_OXA-1_, *catB3*, and *arr-3* genes isolates from dairy farm environment in China

| **Strain ID** | **Antibiotics** | | | | | | | | | | | | | |
| --- | --- | --- | --- | --- | --- | --- | --- | --- | --- | --- | --- | --- | --- | --- |
|  | **AMP** | **CTX** | **RIF** | **C** | **MEM** | **CIP** | **AMK** | **GEN** | **TET** | **TIG** | **SXT** | **FFC** | **CS** | **FOS** |
| 17XJ28 | (R) | (R) | (R) | (R) | (S) | (I) | (S) | (R) | (R) | (S) | (R) | (R) | (S) | (R) |
| 17XJ30 | (R) | (R) | (R) | (R) | (S) | (R) | (S) | (S) | (R) | (S) | (R) | (R) | (S) | (S) |
| 17XJ31 | (R) | (R) | (R) | (R) | (S) | (R) | (S) | (S) | (R) | (S) | (R) | (R) | (S) | (S) |
| 18XJ24 | (R) | (R) | (R) | (R) | (S) | (R) | (S) | (S) | (R) | (S) | (R) | (R) | (S) | (S) |
| 18XJ28 | (R) | (R) | (R) | (R) | (S) | (R) | (R) | (R) | (R) | (S) | (R) | (R) | (S) | (S) |
| 18XJ85 | (R) | (R) | (R) | (R) | (I) | (R) | (R) | (R) | (I) | (S) | (R) | (I) | (S) | (S) |
| 19XJ31 | (R) | (R) | (R) | (R) | (S) | (R) | (R) | (R) | (R) | (R) | (R) | (I) | (S) | (S) |
| R (%) | 100 | 100 | 100 | 100 | 0.00 | 85.7 | 42.8 | 57.1 | 85.7 | 14.3 | 100 | 71.4 | 0.00 | 14.3 |

R= Resistant, S= Susceptible, I= Intermediate. AMP, Ampicillin; CTX, Cefotaxime; MEM, Meropenem; CIP, Ciprofloxacin; AMK, Amikacin; GEN, Gentamicin; TET, Tetracycline; TIG, Tigecycline; SXT, Sulfamethoxazole-Trimethoprim; FFC, Florfenicol; CS, Colistin sulfate; FOS, Fosfomycin

**Multi-drug resistant (MDR) and ESBL-producing strains**

All of the strains were identified as MDR (non-susceptible to at least one antimicrobial agent from three or more antimicrobial classes) and extended-spectrum beta-lactamases (ESBLs) (Table S2).

Table S2. Phenotypic description of MDR *E. coli* harboring *bla*_OXA-1_, *catB3*, and *arr-3* resistance gene isolated from farm environment in China

| Strain ID | No. of antimicrobials | Total antimicrobials | Resistance to no. of antimicrobial classes | Resistance category | Resistance phenotypes | ESBLs | |
| --- | --- | --- | --- | --- | --- | --- | --- |
|  |  |  |  |  |  | CTX | CTX/CLV |
| 17XJ28 | 9 | 14 | 9 | MDR | AMP+CTX+RIF+C+GEN+TET+SXT+FFC+FOS | ≥64 | ≤16 |
| 17XJ30 | 8 | 14 | 8 | MDR | AMP+CTX+RIF+C+CIP+TET+  SXT+FFC | ≥128 | ≤8 |
| 17XJ31 | 8 | 14 | 8 | MDR | AMP+CTX+RIF+C+CIP+TET+  SXT+FFC | ≥64 | ≤16 |
| 18XJ24 | 8 | 14 | 8 | MDR | AMP+CTX+RIF+C+CIP+TET+  SXT+FFC | ≥32 | ≤4 |
| 18XJ28 | 10 | 14 | 9 | MDR | AMP+CTX+RIF+C+CIP+AMK+GEN+TET+SXT+FFC | ≥256 | ≤64 |
| 18XJ85 | 8 | 14 | 7 | MDR | AMP+CTX+RIF+C+CIP+AMK+GEN+SXT | ≥32 | ≤8 |
| 19XJ31 | 10 | 14 | 9 | MDR | AMP+CTX+RIF+C+AMK+GEN+TIG+TET+SXT+FFC | ≥64 | ≤16 |

AMP, Ampicillin; CTX, Cefotaxime; MEM, Meropenem; CIP, Ciprofloxacin; AMK, Amikacin; GEN, Gentamicin; TET, Tetracycline; TIG, Tigecycline; SXT, Sulfamethoxazole-Trimethoprim; FFC, Florfenicol; CS, Colistin sulfate; FOS, Fosfomycin

**Pangenome components**

The genome sizes of the *E. coli* strains co-harboring *bla*_OXA-1_, *catB3*, and *arr-3* ranged from 5.3 Mb to 7.0 Mb with GC content of 50.58 % to 51.55%. The number of annotated genes ranged from 5,114 to 5,815, CRISPR segments from 9 to 90, genomic islands from 1 to 15, and prophages from 9 to 126 (Table S3)

Table S3. Genome component analysis of *E. coli* strains carrying *bla*_OXA-1_, *catB3*, and *arr-3* genes isolates from dairy farm environment in China

| Strain ID | Size (Mb) | GC (%) | Genes | CRISPR | Genomic Islands | tRNA | rRNA | sRNA | Prophages |
| --- | --- | --- | --- | --- | --- | --- | --- | --- | --- |
| 17XJ28 | 5.3 | 51.53 | 5165 | 09 | 12 | 80 | 10 | 68 | 27 |
| 17XJ30 | 5.4 | 51.45 | 5226 | 12 | 14 | 84 | 10 | 64 | 35 |
| 17XJ31 | 6.5 | 50.58 | 5236 | 84 | 01 | 108 | 10 | 76 | 126 |
| 18XJ24 | 5.4 | 51.22 | 5240 | 14 | 15 | 85 | 11 | 75 | 12 |
| 18XJ28 | 5.3 | 51.55 | 5114 | 19 | 08 | 82 | 09 | 65 | 09 |
| 18XJ85 | 7.0 | 50.93 | 5815 | 90 | 01 | 90 | 08 | 86 | 34 |
| 19XJ31 | 5.6 | 51.24 | 5380 | 05 | 14 | 82 | 10 | 69 | 23 |

**Core genome multi-locus sequence typing (cgMLST)**

The cgMLST analysis was determined by cgMLSTFinder 1.2 on the CGE webserver (<https://cge.food.dtu.dk/services/>) based on the total number of allelic loci (2513) to the number of alleles called in core genome database (given in Table S4). The percent alleles identity was set at ≥90%, and it was identified that 4 of the strains, 17XJ28, 17XJ30, 18XJ28, and 19XJ31, fulfilled this criterion and revealed distinct cgSTs, 32937, 129129, 21980, and 119558 respectively. The percent allele identity of 17XJ31, 18XJ24, and 18XJ85 was noted to be 55.51%, 89.61%, and 49.14%, which belong to cgMLST 166368, 148512, and 9438, respectively.

Table S4. Core genome MLST (cgMLST) allelic profiles of *E. coli* strains carrying *bla*_OXA-1_, *catB3*, and *arr-3* genes isolates from dairy farm environment in China

| Strain ID | cgMLST | Total No of allele loci | No of called alleles | % Called alleles | Alleles matches in cgST | % allele identity |
| --- | --- | --- | --- | --- | --- | --- |
| 17XJ28 | 32937 | 2513 | 2380 | 94.71 | 2346 | 93.35 |
| 17XJ30 | 129129 | 2513 | 2381 | 94.75 | 2329 | 92.68 |
| 17XJ31 | 166368 | 2513 | 1759 | 70 | 1395 | 55.51 |
| 18XJ24 | 148512 | 2513 | 2358 | 93.83 | 2252 | 89.61 |
| 18XJ28 | 21980 | 2513 | 2367 | 94.19 | 2342 | 93.2 |
| 18XJ85 | 9438 | 2513 | 1762 | 70.12 | 1235 | 49.14 |
| 19XJ31 | 119558 | 2513 | 2378 | 94.63 | 2315 | 92.12 |

**Phylogenetic tree**

The phylogenic relationship of the seven *bla*_OXA-1_, *catB3*, and *arr-3* co-harboring *E. coli* strains of the present study and other strains retrieved from the NCBI database was determined by phylogeny construction by PHYLOViZ online based on the pubMLST allelic profile. The distance matrix of allelic profiles is given in Table S5.

Table S5. Distance matrix of allelic profiles of this study and NCBI retrieved strains for phylogenetic analysis

| Strain IDs | *adk* | *fumC* | *gyrB* | *icd* | *mdh* | *purA* | *recA* |
| --- | --- | --- | --- | --- | --- | --- | --- |
| EC0430 | 9 | 65 | 5 | 18 | 11 | 8 | 6 |
| SM107 | 6 | 4 | 159 | 44 | 112 | 1 | 17 |
| EC737A1 | 10 | 11 | 4 | 8 | 8 | 8 | 6 |
| EC6563 | 6 | 511 | 4 | 10 | 7 | 8 | 6 |
| EC5 | 10 | 99 | 5 | 91 | 8 | 7 | 2 |
| EBJ003 | 35 | 37 | 29 | 25 | 4 | 5 | 73 |
| ABW_A19 | 168 | 184 | 53 | 140 | 24 | 85 | 42 |
| LD93-1 | 6 | 4 | 1 | 1 | 8 | 1 | 2 |
| E-T207 | 9 | 6 | 33 | 131 | 24 | 8 | 7 |
| 17XJ28 | 43 | 41 | 15 | 18 | 11 | 7 | 6 |
| 17XJ30 | 6 | 19 | 3 | 26 | 11 | 8 | 6 |
| 17XJ31 | 176 | 11 | 4 | 8 | 8 | 8 | 6 |
| 18XJ24 | 6 | 6 | 5 | 10 | 9 | 8 | 6 |
| 18XJ28 | 6 | 4 | 12 | 1 | 20 | 18 | 7 |
| 18XJ85 | 6 | 4 | 12 | 96 | 70 | 468 | 6 |
| 19XJ31 | 92 | 4 | 87 | 96 | 70 | 13 | 2 |

**Mobile genetic elements (MGEs)**

In the present study, we identified MGEs using the ISfinder database (ISfinder (biotoul.fr)) under the CGE server. The total number of MGEs harbored by the seven strains varied from 123 to 222 regardless of sequence similarity and coverage (0-100%). Table S6 summarizes the overall categorical distribution of MGEs, such as small MGEs, gene-carrying MGEs, and conjugative MGEs.

Table S6. Mobile Genetic Elements (MGEs) identified in the core genome of *bla*_OXA-1_, *catB3*, and *arr-3* genes carrying *E. coli* strains recovered from the dairy farm environment in China

| Strain ID | Total | Small MGEs | | MGEs carrying genes (Tns) | | Conjugative MGEs | |
| --- | --- | --- | --- | --- | --- | --- | --- |
| 17XJ28 | 169 | n=123 | MIC=00 | n=45 | UTs=04 | n=01 | CIME=00 |
|  |  |  | MITE=12 |  | CTs=41 |  | IME=01 |
|  |  |  | ISs=111 |  |  |  | ICE=00 |
| 17XJ30 | 123 | n=90 | MIC=00 | n=32 | UTs=07 | n=01 | CIME=00 |
|  |  |  | MITE=11 |  | CTs=25 |  | IME=00 |
|  |  |  | ISs=79 |  |  |  | ICE=01 |
| 17XJ31 | 179 | n=153 | MIC=00 | n=25 | UTs=04 | n=01 | CIME=00 |
|  |  |  | MITE=16 |  | CTs=21 |  | IME=00 |
|  |  |  | ISs=137 |  |  |  | ICE=01 |
| 18XJ24 | 138 | n=104 | MIC=00 | n=34 | UTs=06 | n=00 | CIME=00 |
|  |  |  | MITE=12 |  | CTs=28 |  | IME=00 |
|  |  |  | ISs=92 |  |  |  | ICE=00 |
| 18XJ28 | 222 | n=146 | MIC=00 | n=74 | UTs=11 | n=02 | CIME=00 |
|  |  |  | MITE=12 |  | CTs=63 |  | IME=00 |
|  |  |  | ISs=134 |  |  |  | ICE=02 |
| 18XJ85 | 220 | n=159 | MIC=00 | n=59 | UTs=13 | n=02 | CIME=00 |
|  |  |  | MITE=13 |  | CTs=46 |  | IME=01 |
|  |  |  | ISs=146 |  |  |  | ICE=01 |
| 19XJ31 | 156 | n=103 | MIC=00 | n=52 | UTs=11 | n=01 | CIME=00 |
|  |  |  | MITE=04 |  | CTs=41 |  | IME=01 |
|  |  |  | ISs=99 |  |  |  | ICE=00 |

MIC= Miniature Inverted-Repeat Conjugants; MITE= Miniature Inverted-Repeat Transposable Elements; ISs= Insertion Sequences; Tns= Transposons; UTs= Unit Transposons; CTs= Composite Transposons; CIME= Conjugative Integrative Mobile Elements; IME= Integrative Mobile Elements; ICE= Integrative Conjugative Elements
